# Implementation and utilization of the molecular tumor board to guide precision medicine

**DOI:** 10.18632/oncotarget.18471

**Published:** 2017-06-14

**Authors:** Shuko Harada, Rebecca Arend, Qian Dai, Jessica A. Levesque, Thomas S. Winokur, Rongjun Guo, Martin J. Heslin, Lisle Nabell, L. Burt Nabors, Nita A. Limdi, Kevin A. Roth, Edward E. Partridge, Gene P. Siegal, Eddy S. Yang

**Affiliations:** ^1^ Department of Pathology, University of Alabama at Birmingham, Birmingham, AL, USA; ^2^ Department of Surgery, University of Alabama at Birmingham, Birmingham, AL, USA; ^3^ Department of Medicine, University of Alabama at Birmingham, Birmingham, AL, USA; ^4^ Department of Neurology, University of Alabama at Birmingham, Birmingham, AL, USA; ^5^ Department of Obsterics and Gynecology, University of Alabama at Birmingham, Birmingham, AL, USA; ^6^ Department of Radiation Oncology, University of Alabama at Birmingham, Birmingham, AL, USA; ^7^ Department of The Comprehensive Cancer Center, University of Alabama at Birmingham, Birmingham, AL, USA

**Keywords:** precision medicine, targeted therapy, next generation sequencing, molecular tumor board

## Abstract

**Background:**

With rapid advances in genomic medicine, the complexity of delivering precision medicine to oncology patients across a university health system demanded the creation of a Molecular Tumor Board (MTB) for patient selection and assessment of treatment options. The objective of this report is to analyze our progress to date and discuss the importance of the MTB in the implementation of personalized medicine.

**Materials and Methods:**

Patients were reviewed in the MTB for appropriateness for comprehensive next generation sequencing (NGS) cancer gene set testing based on set criteria that were in place. Because profiling of stage IV lung cancer, colon cancer, and melanoma cancers were standard of care, these cancer types were excluded from this process. We subsequently analyzed the types of cases referred for testing and approved with regards to their results.

**Results:**

191 cases were discussed at the MTB and 132 cases were approved for testing. Forty-six cases (34.8%) had driver mutations that were associated with an active targeted therapeutic agent, including *BRAF, PIK3CA, IDH1, KRAS*, and *BRCA1*. An additional 56 cases (42.4%) had driver mutations previously reported in some type of cancer. Twenty-two cases (16.7%) did not have any clinically significant mutations. Eight cases did not yield adequate DNA. 15 cases were considered for targeted therapy, 13 of which received targeted therapy. One patient experienced a near complete response. Seven of 13 had stable disease or a partial response.

**Conclusions:**

MTB at University of Alabama-Birmingham is unique because it reviews the appropriateness of NGS testing for patients with recurrent cancer and serves as a forum to educate our physicians about the pathways of precision medicine. Our results suggest that our detection of actionable mutations may be higher due to our careful selection. The application of precision medicine and molecular genetic testing for cancer patients remains a continuous educational process for physicians.

## INTRODUCTION

Genomic medicine has been advancing rapidly since the introduction of massive parallel sequencing/next generation sequencing (NGS) technologies. Many commercial vendors as well as academic institutions have been offering extensive molecular testing for cancer care in a clinical setting, including cancer-related gene mutation analysis, copy number variation (CNV), gene rearrangement analysis, and RNA expression signatures [1]. Finding a driver gene mutation can lead to specific targeted therapies, which forms the basis for personalized / precision medicine [2, 3]. NGS is a powerful tool and while the cost associated with the assay is declining, NGS still requires extensive informatics support for data analysis. The integration of these test results into clinical care has been largely left up to the treating physician [4]. The variability among the current available test options and complex results may be confusing to clinicians and pathologists [1]. Furthermore, proper utilization of these assays must be ensured to maximize benefits to the patient while also being cost-effective. In the absence of these standards, efforts to investigate molecular targets may not favorably impact clinical care and potentially could drive up healthcare costs.

The complexity of delivering personalized/precision medicine to oncology patients across a university health system suggested the need to create a Molecular Tumor Board (MTB) three years ago. Unlike MTBs reported elsewhere [5–9], it was decided to discuss the cases before extensive molecular testing to determine whether tumor profiling was appropriate. When the results were available, further treatment options were discussed as a group in the MTB. In this study, we report the three year experience of the MTB at the University of Alabama at Birmingham. The cases discussed at the MTB and those sent for molecular testing along with their results and outcomes are presented.

## RESULTS

### Molecular tumor board (MTB)

**Figure 1:**
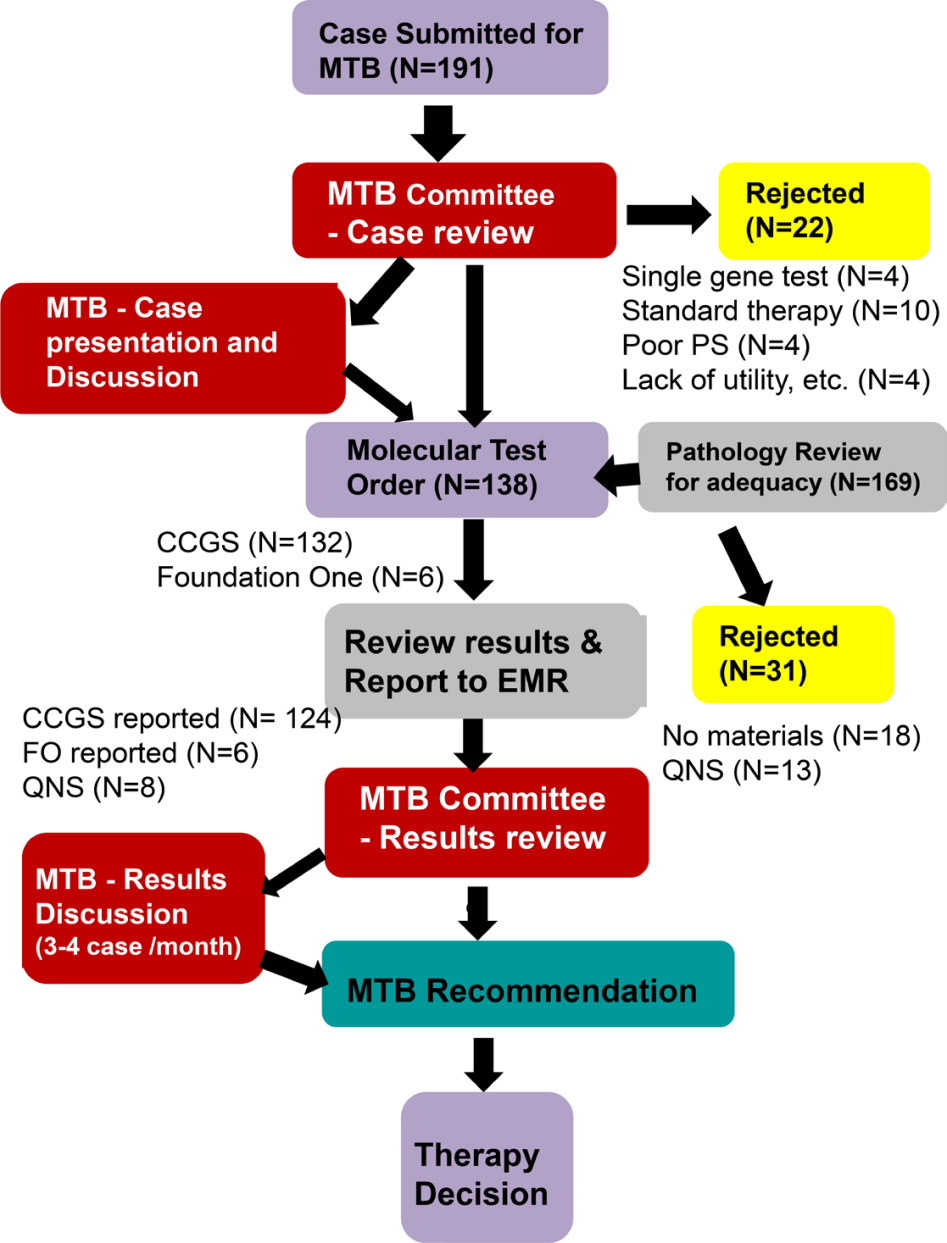
Overview of workflow for molecular tumor board (MTB) CCGS: Comprehensive Cancer Gene Set, FO: Foundation, MTB: Molecular Tumor Board, QNS: Quantity Not Sufficient.

The molecular tumor board (MTB) met monthly for 1 hour. Regular attendees consist of medical oncologists, surgical oncologists, radiation oncologists, surgical pathologists, molecular pathologists, and genetic counselors. Some of these attendees are also physician-scientists, with knowledge of the driver pathways. If needed, basic scientists were invited for further expertise in specific pathways. Ad hoc meetings were also held if clinically warranted. The workflow is illustrated in Figure 1. When a treating physician desired NGS tumor profiling, he/she filled out the request form and submitted it to the MTB executive committee chairs (an oncologist and a molecular pathologist). The committee reviewed the case for appropriateness and the availability of tumor tissue for testing. Selective cases were discussed at the MTB for consensus regarding test approval. After the first year, due to the increased number of cases submitted as well as our experience in gauging appropriateness as a group, tests were approved by the MTB committee on cases that clearly meet the criteria (see below) without discussing the patient at the MTB.

Approved cases were submitted for NGS comprehensive cancer gene set (CCGS) testing. When the results were available, the MTB committee reviewed the results and notified the ordering physician. For selected cases, the results were discussed at the MTB for further treatment decisions as well as for education of our group. Follow-up of clinical outcomes was also performed to track tumor response to therapy.

Before starting the MTB, molecular gene panels were not reimbursed by insurance companies in the state of Alabama. We also did not have a research protocol in place to support genomic sequencing testing as physicians were able to send out cases for testing [4, 8]. This resulted in excess charges to the hospital as well as to the patient. Therefore, we created the MTB as a gatekeeper for appropriateness of patient testing, with Blue Cross Blue Shield of Alabama and UAB Hospital agreeing to reimburse molecular testing that was approved by the MTB at UAB and performed at our academic collaborator, Genomics and Pathology Services (GPS) at Washington University for Comprehensive Cancer Gene Set (CCGS) analysis. Consequently, the vast majority of molecular testing was performed at GPS (https://gps.wustl.edu/) [10, 11]. Testing at other commercial venders [Foundation One (http://foundationone.com) and Caris (http://www.carislifesciences.com)] were also discussed at MTB for some cases when requested.

Initially, criteria were set for comprehensive cancer gene set testing. First, the patient’s neoplasm had to meet one or more of the below five criteria. 1. Treatment resistance or recurrence with no recognized standard of care currently available. 2. A malignant neoplasm of unknown primary / unknown differentiation. 3. Patients with tumors normally treated with surgical resection alone, who experienced multiple tumor recurrences. 4. Patients with a strong family history of cancer or with hereditary syndromes. 5. Patients with tumors of mixed histology or of rare histology. Further questions discussed at the MTB included: 1. If standard therapies were available; 2. If measurable disease was present; 3. If the patient performance status was adequate to wait the 3–4 weeks for results before starting therapy, since the expected overall turnaround time was approximately 4 weeks; 4. If the patient could tolerate the potential therapy; 5. If the cancer type has known driver mutations, such as *EGFR, BRAF, ERBB2 (HER2), ALK*, etc., that were actionable and could be tested by single gene testing - and if so, was the patient treated with the targeted agent and became resistant to those therapies; 6. If there was adequate and appropriate tissue available.

### Cases discussed at MTB

A total of 191 cases were submitted to the MTB (50 male, 141 female; median age 57 years). 132 cases were approved and tested by CCGS (26 in 2013–2014, 66 in 2015 and 40 in January–August, 2016; 2 samples were sent for the same patient because of insufficient DNA quantity on initial testing). Six cases were sent for alternative panel testing that included translocation evaluation (commercial vendor). 53 cases were not tested. Among them, 4 were tested for more specific single gene alteration such as a *COL1A1-PDGFB* fusion [12] by fluorescent *in-situ* hybridization. Thirty-one cases could not be tested because we did not have appropriate/adequate tissue. Ten cases were rejected at the MTB mainly due to the availability of a standard therapy option, and four patients had a poor performance status or went on to rapid supportive care. Four cases were denied due to lack of clinical utility. These include a case with adult type granulosa cell tumor, with high FOXL2 mutaiton rate [13], but this gene was not in CCGS; one case was already previously tested through Caris at an outside institution; one case with rebiopsy of accessible lesion for molecular test showed a different histology (squamous cell carcinoma) compared to the patient’s metastatic/recurrent disease (mucoepidermoid carcinoma); and one pediatric case, which was discussed at the Children’s of Alabama separately.

Typically, overall turnaround time (TAT) is approximately 4 weeks, which includes (1) 3–5 days from the time of request to the specimen being send out, except for rare occasions; (2) 14–28 days with average of 21 days for the laboratory to generate the report, and (3) typically one day for the discussion to be held in MTB. The exceptions for delay include missing pathology materials in the file, need to obtain the pathology materials from outside, need to discuss appropriateness in more detail prior to ordering the test, and the complexity of the results.

There was a steady and significant increase in the number of cases submitted to the MTB in first two years. After the first year, tests were approved on cases that clearly meet the criteria without discussing the patient at the MTB, and discussion focused only on questionable cases and discussion of the molecular test results, treatment plans related to results, and follow-up of the response.

In the last quarter of 2015, the Division of Gynecologic Oncology (GYN) implemented a Precision Medicine Institute (PMI) initiative and created a work-flow within their division to improve the utilization of the MTB. An IRB-approved protocol that included an informed consent and a communication letter to the attending physician summarizing the results of the NGS testing was started. The inclusion criteria was the same as other cases referred to MTB. In the fourth quarter of 2015, 29 GYN cases were requested and 24 of them were tested, accounting for approximately 70% of cases during that period. A similar trend continued in 2016.

**Figure 2:**
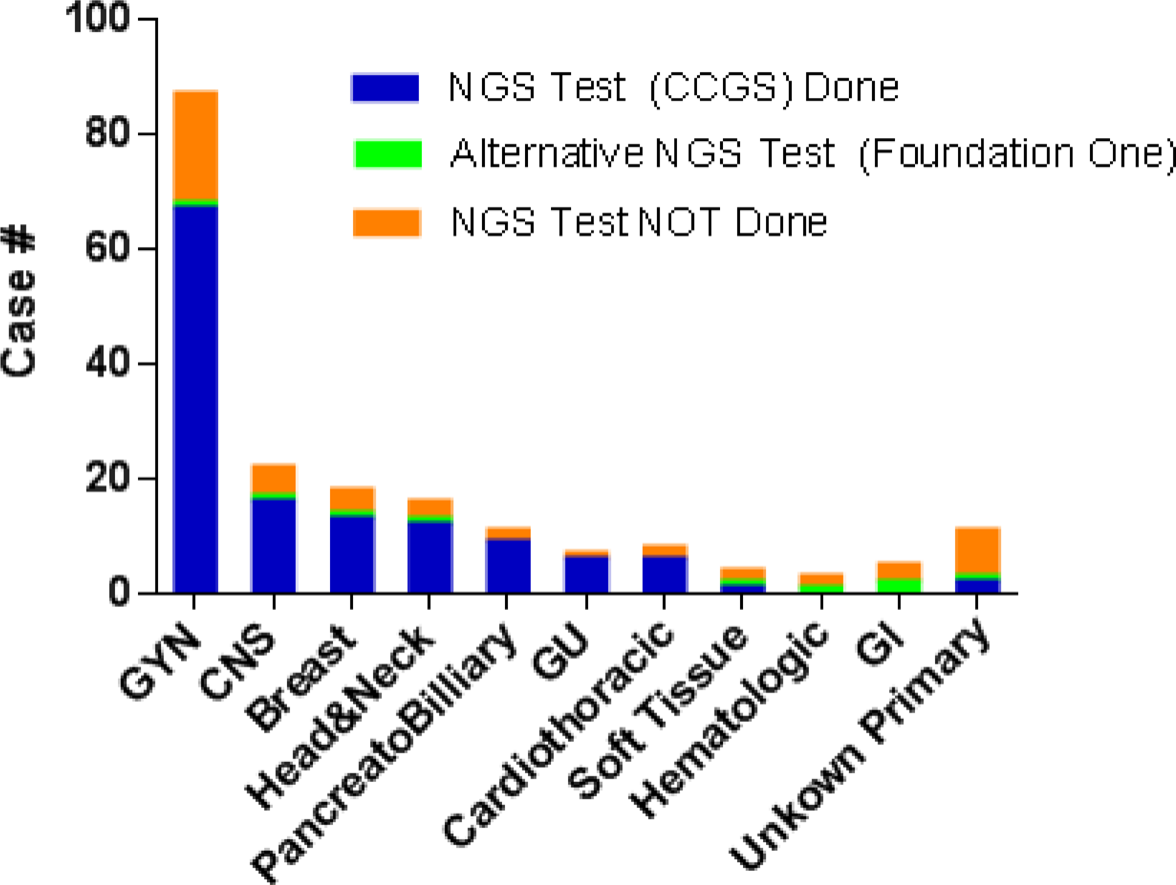
Distribution of tumor types for the cases presented at MTB with decisions NGS Test Performed: Comprehensive Cancer Gene Set; NGS, next generation sequencing; GYN, gynecologic; CNS, central nervous system; GU, genitourinary; GI, gastrointestinal.

Cases have been received from a variety of cancers (Figure 2). GYN tumors are the most common type as described above. The rest are relatively evenly distributed among different types of tumors. The vast majority of the cases (185 of 191, 97%) had metastasis or recurrent disease at MTB presentation. Of note, the “traditional” histologies that have been shown to harbor known actionable driver mutations, such as non-small cell lung, melanoma, and colorectal cancers, were not part of this cohort. These cancers underwent separate single gene testing for these known drivers without MTB approval, as these tests are a part of the standard of care and reimbursable.

### Molecular test results

**Figure 3:**
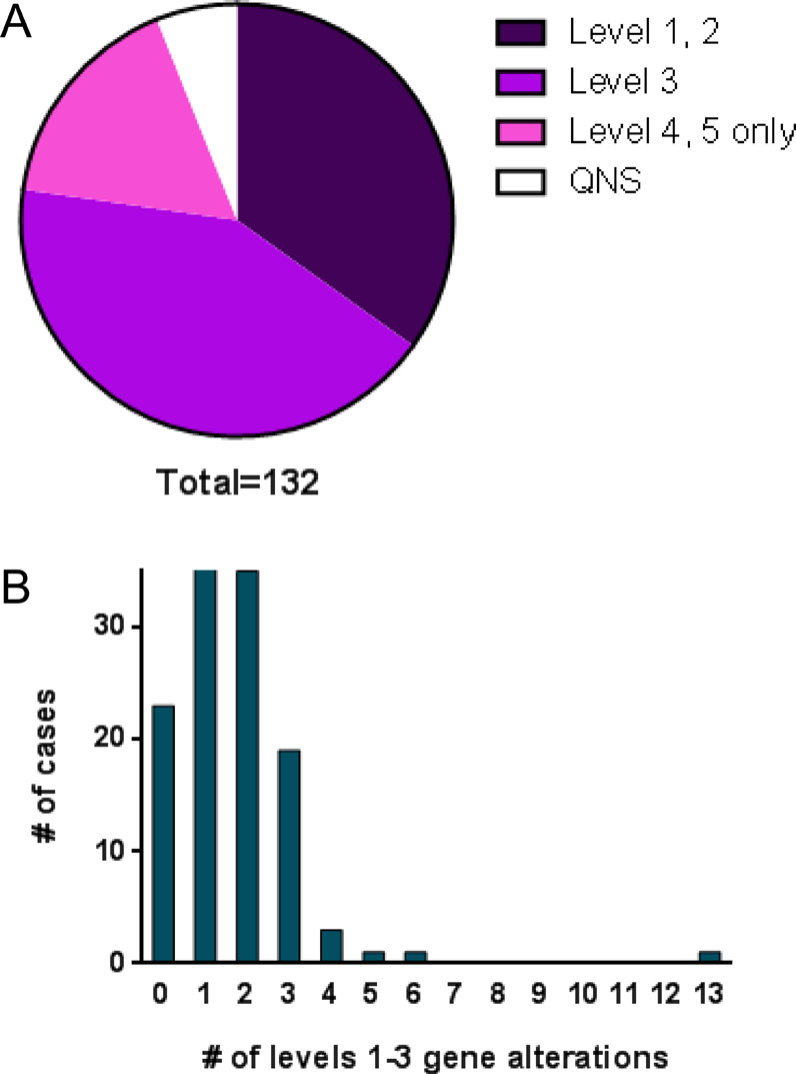
Summary of sequence variations found in cases tested (132 cases, of which 8 cases were QNS) (**A**) Distribution of the results categorized by levels 1–5. QNS, quantity not sufficient. (**B**) Number of patients by the number of levels 1–3 sequence variations.

The variants of the genes in CCSG testing were classified into 5 categories by GPS. Level 1: Predictive or prognostic in tumor type (includes inherited cancer susceptibility variants); Level 2: Predictive or prognostic in other tumor type(s); Level 3: Reported in cancer or other diseases; Level 4: Variant of unknown significance; and Level 5: Known polymorphism. The results of 124 cases were available. Forty-six cases (34.8%) had predictive or prognostic mutations (level 1 or 2) including *BRAF, PIK3CA, IDH1, KRAS, and BRCA1*, many of which were targetable by a therapeutic agent. An additional 56 cases (42.4%) had mutations reported in some type of cancer (level 3). 22 cases (16.7%) did not have any clinically significant mutations (Figure 3A). There were no clear cut differences between the cases with and without significant mutations by tumor location or tumor type (Supplementary Tables 1–3).

**Table 1:** Follow-up summary of the patients with potentially actionable mutations

Treatment Choice	Number of cases	Genes with potentially actionable alteration. (Number in parenthesis indicates number of cases who had same gene mutation)
Targeted therapy	15	
Near complete response	1	*BRAF*
Partial response	2	*PIK3CA (2)*
Stable/mixed	5	*BRAF (2), PIK3CA, BRCA2, BRCA1*
Progressed	5	*BRCA1 (2), PIK3CA, KRAS, BRAF, ESR1*
Stopped due to patients condition	2	*CDKN2A (2)*
Alternative therapy	14	
Chemotherapy, Radiation	7	*KRAS, APC, IDH1 (2), BRCA1 (2), CDKN2A*
Immunotherapy	5	*IDH1, PIK3CA, TP53 (2), BRAF*
Phase I trial	1	*PIK3CA*
Both chemo- and immunotherapy	1	*BRCA1*
Palliative Care	10	*BRCA1 (3), EGFR, IDH2, IDH1, NF1, TERT, PIK3CA, PTEN*
Surveillance	1	*PIK3CA*
No Follow-up data	8	
Followed by outside institution	4	*PIK3CA, KRAS (2), BRCA1*
No information	4	*EGFR, PIK3CA, KRAS (2)*

**Table 2:** The cases received targeted therapy and their outcome

Diagnosis	Mutation	Therapy	Outcome	Detail
High grade Salivary duct carcinoma	*BRAF*p.V600E	BRAF + MEK inhibitor	Near CR	Near complete response over 12 months
Pleomorphic xanthoastrocytoma	*BRAF p*.V600E	BRAF inhibitor	MR/stable	Stable/no improvement for 10 months
Malignant neoplasm in atrium	*BRAF*p.V600E	BRAF inhibitor	MR/stable	Mixed response, then progressed
Glioblastoma (GBM), recurrent	*BRAF*p.V600E	Avastin + BRAF inhibitor + MEK inhibitor	Progress	Questionable response, then progressed and deceased
Metastatic carcinoma of ovary	*BRCA1*c.5277+1 G > A	PARP inhibitor	Progress	Progressed
Clear cell and papillary serous adenocarcinoma	*BRCA1*p.N1236S	PARP inhibitor	MR/stable	Stable for 3 months, then progressed
Papillary serous carcinoma, recurrent	*BRCA2*p.Y1655*	PARP inhibitor	MR/stable	Stable
Metastatic breast carcinoma, HER2 positive	*PIK3CA*p.H1047R	mTOR inhibitor with tamoxifen, trastuzumab, and radiation to the breast	MR/stable	Stable extra-mammary disease for sixteen months, breast disease progressing
Metastatic mucinous adenocarcinoma in lung, consistent with breast primary	*PIK3CA*p.E545K	PI3K inhibitor + cdk4/6 inhibitor + hormonal therapy	PR	Partial response
Metastatic adenocarcinoma in lung, consistent with breast primary	*PIK3CA*p.H1047R	PI3K inhibitor + CDK4/6 inhibitor + hormonal therapy	PR	Partial response
Metastatic breast carcinoma in T9 bone	*ESR1*p.D538G	hormonal therapy, then CDK4/6 inhibitor	Progress	Stable for 1.5 months, then progressed
Endometrioid adenocarcinoma of uterus	*KRAS*p.G12V	MEK inhibitor	Progress	Progressed, then hospice
Adenoid cystic carcinoma, metastasis	*CDKN2A*c.151-2A > T	CDK4/6 inhibitor	Stopped	Not eligible for trial due to an open wound
Metastatic poorly differentiated carcinoma in brain and bone	*CDKN2A*p.R80*	CDK4/6 inhibitor or Phase I trial for MDM2	Sopped	Now hospice
Metastatic carcinoma, history of breast and endometrial carcinoma	PIK3CAp.Q546E	mTOR inhibitor	Progress	Progressed on mTOR inhibitor

(CR: complete response, MR: mixed response, PR; partial response).

The number of gene alterations identified at levels 1–3 ranged between 1 and 6, except for one case that had 13 genes at level 3 (Figure 3B). This latter case was a clear cell carcinoma of the ovary and many variations were found, suggesting hypermutation. Mutation analysis was repeated on a different block and similar results were obtained. Mismatch repair (MMR) protein expression, tested using immunohistochemistry for MLH-1, MSH-2, MSH-6 and PMS2, revealed no evidence of a MMR defect (data not shown), raising the possibility of hypermutation due to mutation of DNA polymerase e (*POLE*) [14, 15].

**Figure 4:**
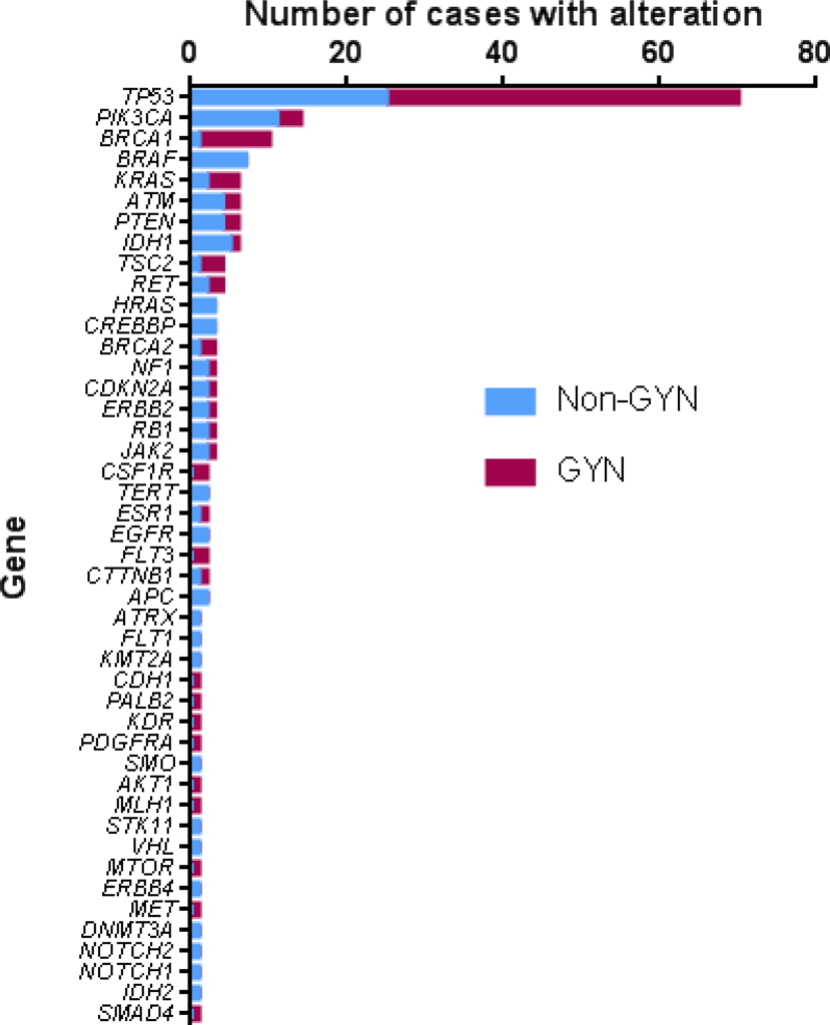
Distribution of the sequence variation by gene GYN – gynecologic tumors, Non-GYN – all others.

The incidence of aberration by gene is shown in Figure 4. Alterations in over 40 different genes were identified. *TP53* alteration was the highest in incidence (70 of 124 patients; 56.5%). Interestingly, mutations found in *TP53* were unique in almost all cases (Supplementary Tables 1 and 2). Notably, more than half of the genes were found to be affected only once, which is consistent with a previous report [5].

Molecular test results of the cases performed by Foundation One instead of CCGS are shown in Supplementary Table 4. Cases 4 and 5 were sent for alternative testing because of the patient and/or family’s request despite the recommendation by the MTB. The results of those cases found *RAS* and *TP53* mutations, which would have been identified by our standard methodologies. Fusion genes or gene amplification were found in cases with a sarcoma diagnosis (cases 1–4), some of which aided in the final diagnosis, but no actionable mutations were identified.

### Follow-up

Forty-eight patients (38.7%) had potentially actionable gene alterations (Supplementary Table 1). Some of the level 3 mutations such as *CDKN2A*, which translates p16 and p14ARF, have a targeted therapy which is currently in a clinical trial; therefore, these cases were included in this list. Sixteen were considered for targeted therapy and 13 received an alternative therapy meaning chemotherapy, radiation therapy or immunotherapy (Table 1). Ten patients were referred to hospice care, eight cases have inadequate follow-up data, and one patient is under surveillance. Choice of targeted therapy options and outcomes are listed in Table 2. Targeted therapy either was not initiated or had to be terminated in three cases due to side effects or other complicating conditions.

Fifteen cases received targeted therapy, and five have progressed on that therapy (mTOR inhibitor, MEK inhibitor, PARP inhibitor, BRAF inhibitor or CDK4/6 inhibitor plus hormonal therapy for a *PIK3CA*, *KRAS*, *BRCA1, BRAF* or an *ESR1* mutation, respectively). Two cases with *PIK3CA* mutated breast cancer had partial responses to a combination of PIK3CA inhibitor, CDK4/6 inhibitor and hormonal therapy. Five cases had stable disease or a mixed response (BRAF inhibitor, mTOR inhibitor or PARP inhibitor for *BRAF*, *PIK3CA* or a *BRCA1/BRCA2* somatic mutation, respectively). One patient with a high grade salivary duct carcinoma with a *BRAF* driver mutation had a near complete response to a combination of BRAF and MEK inhibitors for over 12 months. Notably, six patients, who did not have actionable alterations, received immunotherapy such as anti-PD-1 or anti-PD-L1. However, overall sample size of this cohort is small and the follow-up period is too short to evaluate the effectiveness of biomarker-directed therapies for our patient cohort.

## DISCUSSION

Advances in genomic technologies including NGS have fundamentally altered management of cancer patients. Identification of a driver gene mutation can lead to specific targeted therapies, resulting in personalized /precision medicine [2, 3]. NGS is a powerful tool and the cost associated with the assay is declining. However, the cost is still significant and the test generates massive amounts of information that can be difficult for the clinician to interpret. The integration of these test results into clinical care has been largely left up to the treating physician [4]. In order to better understand the incorporation of NGS into clinical care, the MTB was created three years ago.

Our MTB is unique as compared to ones reported previously [5–9]. First, our MTB has been serving as a “gate-keeper” role in order to avoid unnecessary testing and the associated costs. The patient population presented at the MTB is widely representative of ethnic and racial diversity as well as tumor types but more cases with brain and ovarian cancer have been evaluated here as compared to other published series [5, 6]. Notably, we did not include lung adenocarcinoma, melanoma, or colorectal adenocarcinoma patients in our MTB as single gene tests are routinely performed in-house first on these cases as the standard of care.

As other reports have demonstrated [4–6, 9, 16], and our results confirm, each patient’s malignancy is unique. In our evaluation more than half of the genes were found to be affected once and *TP53* mutation was the most common aberration (56.5%), consistent with other studies [4–6, 16–18]. Interestingly, however, mutations found in *TP53* were unique in almost all cases. Our cohort has a higher proportion of GYN tumors compared to previously reported studies [5, 6]. This finding is due in part to the PMI initiative that was started with an IRB-approved research protocol within the division of gyn oncology.

The most commonly mutated gene, *TP53*, is not currently targetable. Excluding *TP53* mutated cases, the rate of finding actionable mutation in our population is over one third (48 cases; 38.7%) with cancer related gene alterations found in 77% overall. This rate is comparable to previous reported rates in other studies (32–73%), if not significantly higher [4, 5, 9]. Currently, as each study appears to define “actionable” and “significant” mutations differently, direct comparison of “actionable mutation hit rate” is difficult. With standards and guidelines for reporting sequence variants in cancer [19], we believe the measurement would become more universal to be able to compare among institutions. Furthermore, given that our MTB patient population did not include the cancer histologies that have a higher rate of actionable mutations (primary lung adenocarcinoma, melanoma, and colorectal adenocarcinoma), our detection of actionable mutations may be even higher due to our careful case selection. Among patients with potentially actionable mutations who have follow-up data (*n* = 40), 16 patients (40%) were treated with targeted therapy without interruption. This rate is higher than a previously reported study by Tafe, et al. (4 patients; 12.5%) [6] and comparable with more recent study by Bryce et al. (32%) [9]. In GYN tumors, which account for 54% of our cases, alterations in *BRCA1* and *KRAS* were more frequent, whereas *BRAF* and *PIK3CA* mutations were less frequent in these tumors. Advances in pathway-based drug discovery may shift this paradigm to allow more options for targeted therapy in the future.

As part of the MTB, patients were selected for targeted therapy if there was an associated biomarker-directed clinical trial or FDA-approved drug against the driver mutations found for a different indication. Whether patients received the targeted therapy or enrolled on the trial was dependent on patient eligibility (performance status at the time of results) and the oncologist’s experience with off-label use or compassionate treatment. When no variants were found by the CCGS, no additional testing was requested or offered. Treatment of the patient in this situation was limited to either phase I clinical trial, if eligible, or decided by the treating physician.

Our data suggest tumors with a *BRAF* mutation may have the highest chance to respond to a targeted therapy, i.e. BRAF inhibitor, possibly combined with a MEK inhibitor. The finding is consistent with a report on salivary duct carcinoma from Nardi et al. [20] as well as the BASKET study which utilized vemurafenib [21]. Notably, this finding relates to the most common V600E mutation and not other non-codon 600 mutations [21]. Targeted therapies to other driver gene mutations such as *PIK3CA* and somatic *BRCA1* mutations only yielded partial responses or stable disease at best, although our sample size is small draw any conclusions. The majority of our cases showed only a temporary response and eventually progressed on targeted therapy. A possible explanation is that a second driver gene is contributing to the observed resistance. Therapy with multi-agents to block parallel pathways may be necessary to effectively suppress tumor growth. This is evident by the breast cancer with *PIK3CA* mutations who appear to have had a partial response with a combination of PI3K inhibitor, CDK4/6 inhibitor and hormonal therapy.

An additional important aspect of the MTB is its educational component. With the MTB, an opportunity is provided for continuous learning about the application of precision medicine in the genomic era. As seen by the significant increase in the number of the cases discussed at our MTB, we believe the objective of the MTB in its initial years has been met. Currently, we are analyzing the cost-effectiveness of NGS-based efforts in precision medicine and anticipate that further refinement in our cancer gene set testing panels may result in cost-effective molecular genetic testing and improved patient outcomes. Moreover, the number of cases with molecular testing in each institution is limited. A data gathering system with multi-institutional, national or worldwide access that includes treatment and follow-up data in addition to genetic information is needed in order to increase our understanding of the utility of specific agents tied to molecular profiling. An effort to gathering NGS data internationally, initiated by American Association for Cancer Research (AACR), known as AACR Project Genomics, Evidence, Neoplasia, Information, Exchange (GENIE), is very promising [22].

There are some ethical issues related to NGS based testing. As more robust NGS based tests becomes available, the separation between clinical care and research is becoming unclear. It is important to clearly distinguish which components are the standard of the care in both genomic testing and clinical trials [7, 23]. Most likely, the smaller focused panel is more beneficial for the patient with initial cancer diagnosis, especially in early stage [7]. In contrast, broad tumor sequencing would likely to provide value to guide clinical trials and/or off-label use in advanced stage [7], as seen in our MTB populaton. A recent report by Massard et al. [24] demonstrated high throughput genomics improved outcomes, although only 7% of the successfully screened patients benefitted by this approach (MOSCATO 01 Trial).

How to handle incidental findings and possible germline mutations are another issue. The majority of oncology sequencing test was performed on tumor specimens only. However, potential germiline changes, previously reported variants with allele frequency supporting hetero- or homo-zygosity, should be handled with caution, following the published guidelines [25]. Germline testing and genetic counselor consultation is recommended in such cases. Genetic counselors are included in the MTB in our institution. In fact, in ovarian tumors with *BRCA1* mutation, two third of the cases had germline mutation, and they were seen by genetic counselors.

In conclusion, MTB has given us an opportunity for continuous learning about the application of precision medicine and improved patient outcomes. A well-designed MTB will evolve along with the technology to ensure that patients receive the best possible treatment without unnecessary costs or risks, and clinicians derive ongoing educational information to help guide their decisions.

## MATERIALS AND METHODS

### Molecular testing

Blue Cross Blue Shield of Alabama agreed to reimburse for the molecular testing that was approved by MTB at UAB and performed at our academic collaborator, Genomics and Pathology Services (GPS) at Washington University for Comprehensive Cancer Gene Set (CCGS) analysis. Consequently, the vast majority of molecular testing was performed at GPS (https://gps.wustl.edu/) [10, 11].

Upon request, formalin-fixed paraggin embedded tissue blocks were reviewed by molecular pathologists and three 1-mm tissue cores of the area of tumor were obtained to submit for CCGS testing. CCGS uses oligonucleotide-based targeted capture (xGen Lockdown Custom Target Capture Probes, Integrated DNA Technologies, and SeqCap EZ Hybridization and Wash Kit, Roche NimbleGen, Inc.) of whole genome shotgun sequencing libraries (KAPA Hyper Prep Kit and Kapa Library Amplification Kit, KAPA Biosystems, Inc.) [10]. Sequencing of enriched libraries was performed in multiplex on the Illumina HiSeq 2500 using the paired-end, 101 base-pair configuration [10]. The limit of detection of this assay for SNV is 5%, with a 99.1% sensitivity and sensitivity for detecting insertions and deletions of 1–21 bp is 97.7% [10].

Testing at commercial venders [Foundation One (http://foundationone.com) [26] and Caris (http://www.carislifesciences.com)] were also discussed at MTB for some cases when requested. Foundation One uses whole-genome shotgun library construction and hybridization based capture. Sensitivity for SNV is reported to be > 99% at allele frequency of ≥ 10% and for indels of 1–40 bp is 98% at allele frequency of ≥ 20% [26].

### Human subjects

This study was approved by the UAB Institutional Review Board.

## SUPPLEMENTARY MATERIALS TABLES




