# Supplementary material for: Implementation and utilization of the molecular tumor board to guide precision medicine
Source: Oncotarget. 2017 Jun 14;8(34):57845–54. doi: 10.18632/oncotarget.18471 (PMC5593688; doi:10.18632/oncotarget.18471)
Supplement: Supplementary file 1 [file oncotarget-08-57845-s001.pdf]

Implementation and utilization of the molecular tumor board to guide precision medicine

Supplementary Materials

Supplementary Table 1: Cases with potentially clinically actionable genes. See Supplementary_Table_1

Supplementary Table 2: Cases with cancer related gene alterations, but not clinically actionable. See Supplementary_Table_2

Supplementary Table 3: Cases without level 1–3 mutations

Diagnosis	Primary Location
Metastatic carcinoma in brain	Unknown
Primary CNS PNET	Brain
Skull base chordoma	Brain
Pineal parenchymal tumor of intermediate differentiation	Brain
Metastatic breast cancer	Breast
Sarcoma with histiocytic differentiation in breast	Breast
Hurtle cell carcinoma, metastatic	Thyroid
Metastatic adenoid cystic carcinoma	Salivary gland
Metastatic adenoid cystic carcinoma	Salivary gland
Adenoid cystic carcinoma	Salivary gland
Clear cell papillary renal cell carcinoma	Kidney
Upper urinary tract squamous cell carcinoma	Urinary tract
Poorly differentiated carcinoma, favor small cell	Pancreas
Papillary serous adenocarcinoma	GYN
Recurrent granulosa cell tumor	GYN
Carcinosarcoma of ovary, metastasis to brain	GYN
Neuroendocrine tumor, uterine origin	GYN
Endometrioid carcinoma, metastatic	GYN
Leiomyosarcoma, recurrent	GYN
Papillary serous carcinoma, recurrent	GYN
Papillary serous carcinoma, recurrent	GYN
Endometrial adenocarcinoma to colon	GYN

Supplementary Table 4: Cases tested for foundation one

	Age, Sex	Diagnosis	Foundation One Results
**1**	33F	Spindle cell neoplasm with metastasis	*KRAS* amp, *CCND2* amp, *CDK4* amp, *MDM2* amp, *FGF23* amp, *FGF*6 amp, *KDM5A* amp
**2**	45M	Myxoid liposarcoma vs extraskeletal myxoid chondrosarcoma	*EWSR1* fusion; *PIK3CA* mutation (subclone)
**3**	40F	Metastatic follicular dendritic cell sarcoma	*HDAC1* E396D, *TP53* R248W, *EWSR1-OSM* fusion, *SETBP1* G870D
**4**	56F	Pituitary adenoma, recurred path shows rhabdomyosarcoma	*FGFR*1 amp, *MYC* amp, *MYST3* amp, *ZNF703* amp
**5**	60F	Papillary serous ca with metastasis, recurrent	*KRAS* G12V, *TP53* P278H
**6**	54F	Triple negative breast cancer, now metastasis to lung, liver and brain	*HRAS* G12S, *PIK3CA* E545N, N1044S, *CCND3* amp, *MYC* amp (equivocal), *TP53* A79fs*44, *RB1* N328fs*4

F, female; amp = amplification.
