# Supplementary material for: Implementation and utilization of the molecular tumor board to guide precision medicine
Source: Oncotarget. 2017 Jun 14;8(34):57845–54. doi: 10.18632/oncotarget.18471 (PMC5593688; doi:10.18632/oncotarget.18471)
Supplement: Supplementary file 2 [file oncotarget-08-57845-s002.docx]

Supplemental Table 1. Cases with Potentially Clinically Actionable Genes

| Diagnosis | Primary Location | Level 1 | Level 2 | Level 3 |
| --- | --- | --- | --- | --- |
| Pleomorphic xanthoastrocytoma | Brain | *BRAF* p.V600E, TP53 p.R158G |  | *DNMT3A* p.E30A |
| High grade Salivary duct carcinoma | Salivary gland | *BRAF* p.V600E |  |  |
| High grade Salivary duct carcinoma (recurrent after BRAF inhibitor treatment) | Salivary gland | *BRAF* p.V600E |  |  |
| Malignant neoplasm in atrium | Heart | *BRAF* p.V600E |  | *TP53* p.P118L, *RB1* p.R787* |
| Glioblastoma (GBM), recurrent | Brain |  | *BRAF* p.V600E |  |
| Recurrent Glioblastoma (GBM) | Brain | *EGFR* p.A289V, *PIK3CA* p.E545K |  | *TP53* p.E258K |
| GBM transformed from grade II | Brain | *IDH1* p.R132H |  | *PTEN* p.R15K, *TP53* p.H193Y, V173M |
| Recurrent diffuse astrocytoma grade II | Brain | *IDH1* p.R132H |  | *ATRX* p.F2113Sfs*9, *TP53* p.G266E |
| Multi-recurrent GBM | Brain | *IDH1* p.R132H |  | *TP53* p.I255_I255del |
| GBM, recurrent | Brain | *TERT* c.-124C>T |  | *EGFR* p.A289V |
| GBM, recurrent | Brain |  | *TERT* c.-146C>T | *PTEN* p/W111*; *TP53* p.G245R |
| Pilocytic astrocytoma with atypical feature and high Ki67, recurrent | Brain | *NF1* p.K583R; *TP53* p.P301Rfs*4 |  |  |
| Metastatic adenocarcinoma of ovary | GYN | *BRCA1* c.5277+1 G>A |  | *NF1* p.R1362*; *TP53* p.R213Dfs*34 |
| Adenocarcinoma of ovary, primary serous papillary with clear cell | GYN | *BRCA1* p.R1679Kfs*3 | *TP53* p.R248W |  |
| Papillary serous carcinoma, recurrent | GYN | *BRCA1 p*.Q1756Pfs*74 |  | *TP53* p.Y220C, *TSC2* p.A357V |
| Papillary serous carcinoma | GYN | *BRCA1* p.R1992* |  | *TP53* p.C135Y |
| Papillary serous carcinoma in ileocecum | GYN | *BRCA1* p.D825Efs*21 |  |  |
| Clear cell and papillary serous adenocarcinoma | GYN |  |  | *BRCA1* p.N1236S, *TP53* c.376-2_378delinsT |
| Metastatic papillary serous ca | GYN |  | *BRCA1* p.V757Ffs*8 | *MET* p.R988C, *TP53* p.I195N |
| Papillary serous carcinoma, recurrent | GYN | *BRCA1* p.E720*/E673* |  | *TP53* c.920-2A>G |
| Carcinosarcoma / MMMT | GYN | *BRCA1* p.Q1756* |  | *TP53* p.M237K |
| Papillary serous carcinoma, recurrent | GYN | *BRCA2* p.Y1655* |  | *TP53* p.C242* |
| Endometrial adenocarcinoma with squamous differentiation in ovary | GYN | *PTEN* p.R130Q; *PIK3CA* p.R88Q, R93Q; *CTNNB1* p.G34E |  |  |
| Endometrioid adenocarcinoma | GYN | *PTEN* p.V175L |  | *TP53* p.E221* |
| Metastatic papillary serous carcinoma | GYN | *TP53* p.R116Q |  | *PIK3CA* p.M1043V, *RB1* p.358Q |
| High grade serous carcinoma, recurrent | GYN | *PIK3CA* p.K111E |  | *TP53* p.R248Q |
| Adrenal cortical carcinoma | Adrenal gland | *APC* p.Q625*, *PIK3CA* p.H1047R (3%) |  | *JAK2* p.R1063H |
| High grade salivary duct adenocarcinoma | Salivary gland | *PIK3CA* p.H1047R | *HRAS* p.Q61R, *BRAF* p.G466V |  |
| Metastatic breast carcinoma | Breast | *PIK3CA* p.H1047R |  | *TP53* p.S166* |
| Metastatic adenocarcinoma in liver, breast primary | Breast | *PIK3CA* p.E545K |  | *TP53* p.C176S |
| Breast carcinoma, metastatic, triple negative | Breast | *PIK3CA* p.H1047R, *TP53* p.C242Afs*5 |  | *HRAS* p.G12S |
| Metastatic mucinous adenocarcinoma in lung, consistent with breast primary | Breast | *PIK3CA* p.E545K |  | *CREBBP* p.V777L*, ERBB2* p.G727A*, TSC2* p.A583T |
| Metastatic adenocarcinoma in lung, consistent with breast primary | Breast | *PIK3CA* p.H1047R |  | *ERBB2* p.S310Y |
| Metastatic breast carcinoma | Breast | *TP53* p.R248Q | *RET* p.R982C |  |
| Metastatic breast carcinoma in T9 bone | Breast | *ESR1* p.D538G |  |  |
| Cholangiocarcinoma | Hepatobiliary |  | *IDH1* p.132C | *ATM* p.D1853V |
| Cholangiocarcinoma | Hepatobiliary |  | *IDH2* p.R172K |  |
| Metastatic pancreas adenocarcinoma | Hepatobiliary |  | *KRAS* p.Q61H | *NOTCH2* p.P6Rfs*27, *RET* p.R982C |
| Metastatic adenocarcinoma in the lung, pancreas origin | Hepatobiliary |  | *KRAS* p.G12D | *ATM* p.V410A |
| Endometrioid adenocarcinoma | GYN |  | *KRAS* p.G12V |  |
| Endometrioid adenocarcinoma of uterus | GYN |  | *KRAS* p.G12V | **Microsatellite instable |
| Adenocarcinoma of ovary, mixed papillary serous and endometrioid | GYN |  | *KRAS* p.G12V | *TP53* p.A159V, *JAK2* p.G571S, *MLH1* p.K618A |
| Papillary serous carcinoma, recurrent | GYN |  | *KRAS* p.G12D |  |
| Adrenal corticoid carcinoma | Adrenal gland |  | *TP53* p.R273C, *CTNNB1* p.S45P |  |
| Adenoid cystic carcinoma, metastatic | Head & Neck |  |  | *CDKN2A* c.151-2A>T |
| Metastatic poorly differentiated carcinoma in brain/bone | Brain |  |  | *CDKN2A* R80* |
| Papillary serous carcinoma of ovary | GYN |  |  | *CDKN2A* p.R58* |
| Metastatic carcinoma, history of breast and endometrial carcinoma | Unknown |  |  | *PIK3CA* p.Q546E |

Supplemental Table 2. Cases with cancer related gene alterations, but not clinically actionable

| Diagnosis | Primary Location | Genes |
| --- | --- | --- |
| Malignant solitary fibrous tumor | Lung/Pleura | *TP53* p.P250L |
| Poorly differentiated carcinoma in lung | Lung | *TP53* p.A27Hfs*26, S127F, *APC* p.P1634L, R2326*, *KMT2A (MLL)* p.S476F, R862* |
| Poorly differentiated malignancy in lung, recurred | Lung | *TP53* p. H179R |
| Adenoid cystic carcinoma of pleura | Lung/Pleura | *HRAS* p.G13R |
| Malignant germ cell tumor | Mediastinum | *TP53* p.H179R |
| Adenoid cystic carcinoma, recurrent | Salivary gland | *ATM* p.D1853V, *JAK2* p.N1108S |
| Adenoid cystic carcinoma | Salivary gland | *TP53* p.A27V, *ATM* p.S707P, *ERBB4* p.E755K, *BAP1* p.I455Mfs*127 |
| Ex-pleomorphic adenocarcinoma | Salivary gland | *PTEN* p.V166Sfs*14, *CDH1* c.1565+1G>A |
| Squamous cell carcinoma of ear canal | Head & Neck | *TP53* p.L348*, *RB1* p.358* |
| Malignant glioma | Brain | *TP53* c.376-2A>G |
| Meningioma, grade III | Brain | *SMO* p.G16_L17insL |
| Metastatic cholangiocarcinoma | Hepatobiliary | *BRAF* p.D594N, *TP53* p.E286K |
| Metastatic renal cell carcinoma, clear cell type | Kidney | *VHL* p.L118Efs*39 |
| Adrenocortical carcinoma, metastatic | Adrenal gland | *TP53* p.T211Ffs*4; *NF1* p.S665F |
| Malignant myoepithelial lesion | Soft tissue | *NOTCH1* p.I2550V, *PIK3CA* p.S673T, *PTEN* p.E43Kfs*11 |
| Invasive ductal carcinoma, triple negative, recurrent | Breast | *BRCA1* p.T790A, p.R794W, *BRCA2* p.I3412V; *FLT1* p.T568M; *PIK3CA* p.E545G; *TP53* p.A144G |
| High grade sarcoma of breast | Breast | *STK11* p.F354L, *TP53* p.R273H (2%) |
| Invasive ductal carcinoma, metastasis to liver | Breast | *CREBBP* p.V1924M |
| Invasive ductal carcinoma, triple negative | Breast | *TP53* c.673-1G>T |
| High grade endometrial stromal sarcoma with smooth muscle differentiation | GYN | *TP53* p.G245S |
| Metastatic carcinoma, ovarian origin | GYN | *TP53* p.V216M |
| Ovarian adenocarcinoma, mixed clear cell and papillary serous | GYN | *TP53* p.V272M |
| Papillary serous adenocarcinoma, recurrent | GYN | *TP53* p.R282W |
| Papillary serous adenocarcinoma, metastatic | GYN | *TP53* c.559+1G>C |
| Metastatic high grade adenocarcinoma, ovarian origin | GYN | *TP53* p.K132T, *CDH1* p.G62S |
| Papillary serous adenocarcinoma, recurrent | GYN | *TP53* p.W91Gfs*32 |
| High grade Adenocarcinoma of ovary in ileum | GYN | *TP53* p.V272Afs*33 |
| Papillary serous carcinoma, metastatic and recurrent | GYN | *TP53* p.I195T |
| Papillary serous carcinoma, metastatic to liver | GYN | *TP53* p.R306*, *MTOR* p.M2371I |
| Papillary serous adenocarcinoma | GYN | *TP53* p.C275F; *CSF1R* p.V32G |
| Metastatic papillary serous carcinoma | GYN | *TP53* p.H179R, *AKT* p.E17K, *BRCA2* p.V2728I, *ERBB2* p.I624V |
| Papillary serous carcinoma | GYN | *TP53* c.376-1G>A |
| Metastatic papillary serous carcinoma | GYN | *TP53* p.R342*; *ATM* p.S49C |
| Metastatic papillary serous carcinoma | GYN | *TP53* p.H179L |
| Metastatic papillary serous carcinoma | GYN | *TP53* p.G245Afs*2 |
| Metastatic papillary serous carcinoma | GYN | *TP53* p.M246T |
| Papillary serous carcinoma, recurrent | GYN | *FLT3* p.V194M; *TP53* p.G245D |
| Clear cell carcinoma of ovary | GYN | *TP53* p.E285* |
| Metastatic carcinosarcoma/MMMT | GYN | *TP53* p.C141fs*29 |
| Papillary serous carcinoma of ovary | GYN | *TP53* p.R174Sfs*67*; ATM* R2459C |
| Papillary serous carcinoma with focal clear cell differentiation | GYN | *TP53* p.R273C; *CSF1R* p.R106W |
| Papillary serous carcinoma, recurrent | GYN | *TP53* p.C176Y; *TSC2* p.R1706C |
| Papillary serous carcinoma, recurrent | GYN | *TP53* p.Y163C |
| Papillary serous carcinoma, recurrent | GYN | *TP53* p.Y220C |
| Papillary serous carcinoma, recurrent | GYN | *TP53* p.P295Lfs*50*; KDR* p.T1152M |
| Papillary serous carcinoma, recurrent | GYN | *TP53* p.R117S*; ESR1* p.H6Y; *PALB2* p.L939W |
| Papillary serous adenocarcinoma with endometrioid differentiation | GYN | *TP53* p.I123N |
| Papillary serous adenocarcinoma with focal endometrioid differentiation | GYN | *TP53* p.S241C |
| HG Papillary serous carcinoma, metastatic | GYN | *TP53* p.R81*/R213* |
| Papillary serous carcinoma of ovary with metastasis to colon | GYN | *RET* P841L*; TP53* C135Y |
| Adenocarcinoma of ovary, recurrent | GYN | *TSC2* c.4850_4867+16del; *TP53* p.C141W |
| High grade leiomyosarcoma | GYN | *RET* p.R982C, *SMAD4* p.I525G |
| Metastatic papillary serous carcinoma | GYN | *CDH1* p.A592T |
| Clear cell carcinoma of ovary | GYN | 13 level 3 variants detected |
